# Based on CT at the third lumbar spine level, the skeletal muscle index and psoas muscle index can predict osteoporosis

**DOI:** 10.1186/s12891-022-05887-5

**Published:** 2022-10-24

**Authors:** Cheng-bin Huang, Duo-duo Lin, Jian-qiang Huang, Wei Hu

**Affiliations:** 1 Department of Orthopaedic Surgery, The Second Affiliated Hospital and Yuying Childrens Hospital of Wenzhou Medical University, Wenzhou, 325000 China; 2 Key Laboratory of Orthopaedics of Zhejiang Province, Wenzhou, 325000 China; 3 The Second Affiliated Hospital and Yuying Childrens Hospital of Wenzhou Medical University, Wenzhou, 325000 China

**Keywords:** Osteoporosis, Skeletal muscle index, Psoas muscle index, Computed tomography, Bone mineral density

## Abstract

**Background:**

With the increasing number of studies on osteoporosis and muscle adipose tissue, existing studies have shown that skeletal muscle tissue and adipose tissue are closely related to osteoporosis by dual-energy x-ray absorptiometry (DXA) measurement. However, few studies have explored whether the skeletal muscle and adipose tissue index measured at the lumbar spine 3 (L3) level are closely related to bone mineral density (BMD) and can even predict osteoporosis. Therefore, this study aimed to prove whether skeletal muscle and adipose tissue index measured by computed tomography (CT) images based on a single layer are closely related to BMD.

**Methods:**

A total of 180 participants were enrolled in this study to obtain skeletal muscle index (SMI), psoas muscle index (PMI), subcutaneous fat index (SFI), visceral fat index (VFI), and the visceral-to-subcutaneous ratio of the fat area (VSR) at L3 levels and divide them into osteoporotic and normal groups based on the T-score of DXA. Spearman rank correlation was used to analyze the correlation between SMI, PMI, SFI, VFI, VSR, and BMD. Similarly, spearman rank correlation was also used to analyze the correlation between SMI, PMI, SFI, VFI, VSR, and the fracture risk assessment tool (FRAX). Receiver operating characteristic (ROC) was used to analyze the efficacy of SMI, PMI, SFI, VFI, and VSR in predicting osteoporosis.

**Results:**

BMD of L1-4 was closely correlated with SMI, PMI, VFI and VSR (r = 0.199 *p* = 0.008, r = 0.422 *p* < 0.001, r = 0.253 *p* = 0.001, r = 0.310 *p* < 0.001). BMD of the femoral neck was only correlated with PMI and SFI (r = 0.268 *p* < 0.001, r = − 0.164 p-0.028). FRAX (major osteoporotic fracture) was only closely related to PMI (r = − 0.397 *p* < 0.001). FRAX (hip fracture) was closely related to SMI and PMI (r = − 0.183 *p* = 0.014, r = − 0.353 *p* < 0.001). Besides, FRAX (major osteoporotic fracture and hip fracture) did not correlate with VFI, SFI, and VSR. SMI and PMI were statistically significant, with the area under the curve (AUC) of 0.400 (95% confidence interval 0.312-0.488 *p* = 0.024) and 0.327 (95% confidence interval 0.244-0.410 *p* < 0.001), respectively. VFI, SFI, and VSR were not statistically significant in predicting osteoporosis.

**Conclusions:**

This study demonstrated that L3-based muscle index could assist clinicians in the diagnosis of osteoporosis to a certain extent, and PMI is superior to SMI in the diagnosis of osteoporosis. In addition, VFI, SFI, and VSR do not help clinicians to diagnose osteoporosis well.

## Introduction

With the world’s population aging, osteoporosis and osteoporotic fractures have become one of the leading causes of mortality in the elderly. Osteoporosis is a systemic disease in which bone mass is reduced, and the microstructure of bone is damaged, so the strength of bone is decreased, and fractures are easy to occur [1, 2]. According to European clinical guidelines, the gold standard for the diagnosis of osteoporosis is a bone mineral density (BMD) T score of less than − 2.5 on the femoral neck or lumbar spine as measured by dual-energy x-ray absorptiometry (DXA) [3]. At present, the treatment of osteoporosis is still based on anti-osteoporosis drugs such as bisphosphonates and denosumab [4]. However, with the increase of studies on the relationship between muscle and osteoporosis, some studies suggest that osteoporosis patients exercise to strengthen muscle mass to prevent and treat osteoporosis [4, 5]. In addition, it has been found that low muscle mass is a significant risk factor for falls in patients with osteoporosis, thus increasing the probability of osteoporotic fracture [6].

Sarcopenia is a syndrome caused by the continued loss of skeletal muscle mass, strength, and function [7]. As adults age 40, skeletal muscle mass decreases by about 1% per year [8]. According to the expert consensus, skeletal muscle index (SMI) measured by DXA is the primary diagnosis of sarcopenia [9]. In addition, adipose tissue is closely related to muscle and bone tissue. When an excessive increase in fat accompanies the deterioration of bone and muscle tissue, it is called osteosarcopenic obesity [10, 11]. Furthermore, as with muscle tissue, DXA is the primary tool for measuring adipose tissue. However, some studies have proved that only one lumbar spine 3 (L3) computed tomography (CT) image can well reflect the skeletal muscle index and adipose tissue index of the whole body (the area of muscle or adipose tissue at the L3 level divided by the square of height) [12].

With the increasing number of studies on osteoporosis and muscle adipose tissue, existing studies have shown that skeletal muscle tissue and adipose tissue are closely related to osteoporosis by DXA measurement [13, 14]. However, few studies have explored whether the skeletal muscle and adipose tissue index measured at the L3 level are closely related to BMD and can even predict osteoporosis. Therefore, this study aimed to prove whether skeletal muscle and adipose tissue index measured by CT images based on a single layer are closely related to BMD, thus providing an auxiliary means for clinicians to diagnose osteoporosis.

## Methods

### Study population

With the institutional review committee’s approval, we retrospectively collected patients older than 40 who underwent DXA and abdominal CT examinations from the Second Affiliated Hospital of Wenzhou Medical University database from January 2017 to January 2021. The inclusion criteria were:1) The interval between unenhanced abdominal CT and DXA (lumbar spine and femoral neck) was less than 3 months, and 2) Age ≥ 40 years. The exclusion criteria were: 1) No unenhanced abdominal CT and DXA and 2) the presence of a lumbar osteolytic lesion, lumbar spine surgery, scoliosis, dementia, delirium, or other conditions that made completing questionnaires difficult.

### Skeletal muscle and fat index measurements

**Fig. 1:**
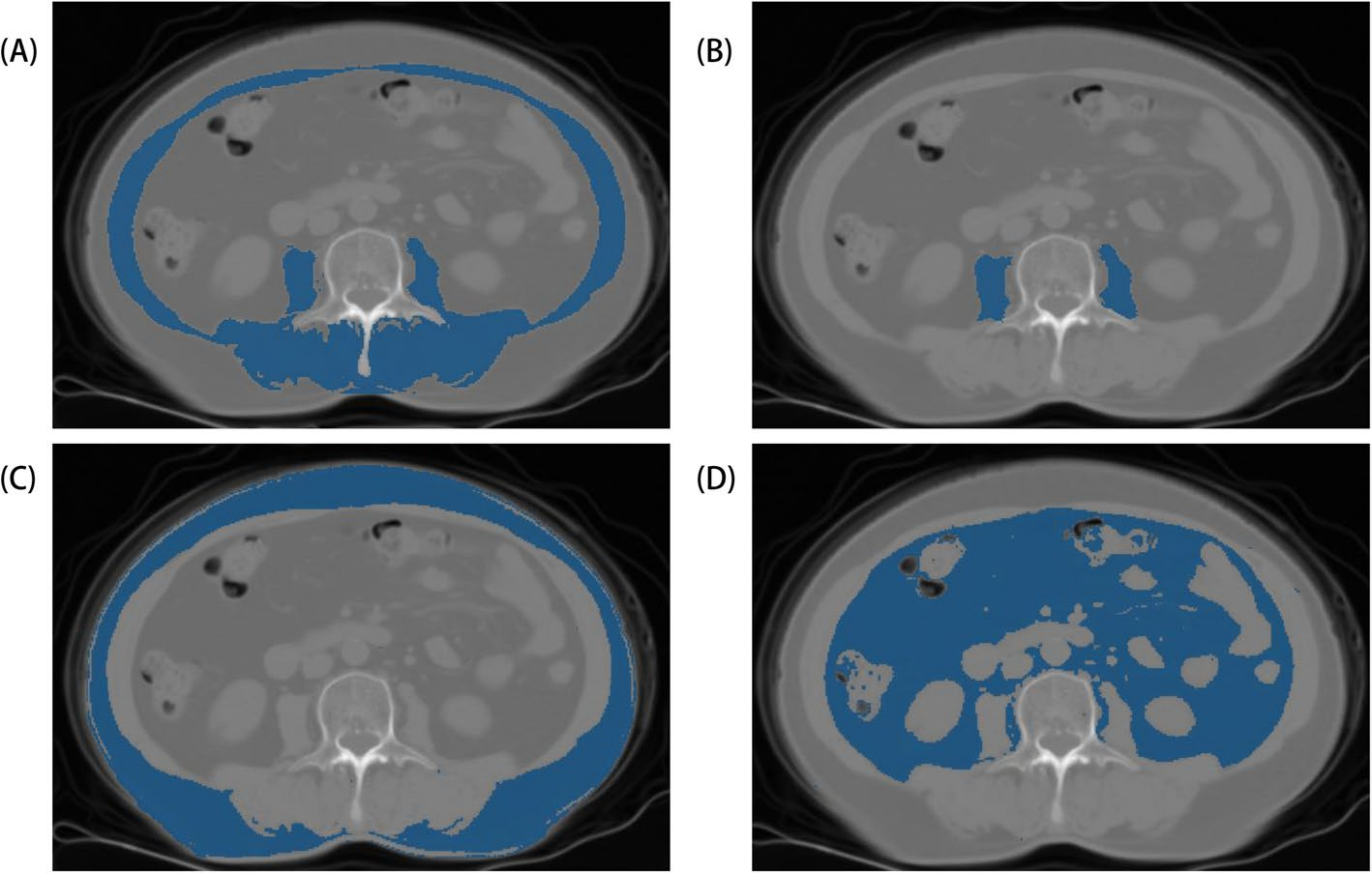
Measurement of the skeletal muscle index (**A**), psoas muscle index (**B**), subcutaneous fat index (**C**) and visceral fat index (**D**) using computed tomography at the L3 level

Abdominal CT data were obtained by the picture archiving and communication system (Philips) operated at 120 kV and 250 mA with a slice thickness of 5 mm. In addition, the CT data were obtained after the DXA examination within 3 months. Image J (NIH Image J version 1.52c) software was used to measure the cross-sectional area of skeletal muscle, psoas muscle, subcutaneous fat, and visceral fat at the horizontal plane of the L3 vertebral body midsection (Fig. 1). According to previous studies [15], the threshold of skeletal muscle is -29HU ~ 150HU, and the adipose tissue threshold is -190HU ~ −30HU. This study measured the skeletal muscle area, psoas muscle area, subcutaneous fat area, and visceral fat area. Obtained area values were divided by the square of the patient’s height (m^2^) to get skeletal muscle index (SMI), psoas muscle index (PMI), subcutaneous fat index (SFI), and visceral fat index (VFI). The visceral-to-subcutaneous ratio of the fat area (VSR) was also calculated. Two experts have more than 5 years of clinical work experience and are skilled in using Image J software. One of the experts outlined skeletal muscle, psoas muscle, subcutaneous fat, and visceral fat on CT images. Another expert checked the results of the contours.

### BMD, diagnosis of osteoporosis and fracture risk assessment tool (FRAX)

BMD was measured by DXA (Lunar Prodigy Advance) at the L1, L2, L3, L4, entire lumbar (L1-4), and femoral neck. T scores of the whole lumbar region and femoral neck were evaluated. According to European clinical guidelines [3], patients with a lumbar (L1-4) or femoral neck T-score of less than − 2.5 are diagnosed with osteoporosis. Besides, the FRAX survey was obtained by face-to-face or telephone communication with patients. Gender, age, height, weight, prior fragility fracture, parental hip fracture, systemic glucocorticoid use, rheumatoid arthritis, other cases of secondary osteoporosis, excess alcohol intake, smoking, systemic glucocorticoid use, rheumatoid arthritis, and femoral neck of BMD were included. Log in to the Chinese version of https://www.sheffield.ac.uk/FRAX/?lang=chs of the FRAX model, inputting the baseline data of patients. The BMD of the femoral neck was included in the FRAX model to calculate the 10-year fracture probability (mainly osteoporotic and hip fractures).

### Statistics

Data distribution was tested using the Shapiro-Wilk test. As appropriate, patient characteristics were described using median (interquartile range [IQR]) and mean ± standard deviation, frequency, and percentage. A nonparametric test (Mann-Whitney U test or Kruskal-Wallis test) was applied for data with non-normal distribution or heterogeneity of variances. Categorical variables were expressed as percentages and analyzed using the Pearson Chi-squared test. Spearman rank correlation was used to determine the correlation between BMD and skeletal muscle and fat index (SMI, PMI, VFI, SFI, and VSR). Spearman rank correlation was also used to determine the correlation between FRAX (major osteoporotic fracture and hip fracture) and skeletal muscle and fat index. When the absolute value of the coefficient of Spearman’s rank correlation is closer to 1, the correlation between the two variables is more robust [16]. In addition, the receiver operating characteristic (ROC) curve was used to determine the effectiveness of these indices in predicting osteoporosis. All statistics were calculated using SPSS software (version 26.0; SPSS Inc., Chicago, IL, USA).

## Results

### Patient characteristics

The characteristics of included patients are presented in Table 1. A total of 180 participants were included in the study, including 112 in the normal group and 68 in the osteoporosis group. There were statistically significant differences in age and height between the two groups (63 versus 65 *p* = 0.001; 160 versus 157 *p* = 0.026). There were no significant differences in body weight, BMI (body mass index), sex, hypertension, diabetes, hyperlipidemia, smoking, alcohol consumption, NRS (nutritional risk screening) 2002 score [17], and Barthel index [18] between the two groups. Besides, Table 2 presents the prevalence of FRAX-related factors.

**Table 1:** Comparison of clinical characteristics between normal group and osteoporosis group

	Normal (112)	Osteoporosis(68)	*P* value
Age (years)	63 ± 9	65 ± 13	0.001
Height (cm)	160 (155-168)	157 (153-165)	0.026
Weight (kg)	62.36 ± 8.95	58.24 ± 10.57	0.307
BMI (kg/m^2^)	23.98 ± 3.03	23.06 ± 3.67	0.396
Gender			0.075
Female, n(%)	66 (58.9)	49 (75.1)	
Male, n(%)	46 (41.1)	19 (27.9)	
Hypertension, n(%)	72 (64.3)	36 (52.9)	0.132
Diabetes, n(%)	82 (73.2)	52 (76.5)	0.627
Hyperlipidemia, n(%)	47 (42.0)	30 (44.1)	0.777
Current smoking, n(%)	19 (17.0)	7 (10.3)	0.217
Excess alcohol intake, n(%)	12 (10.7)	8 (11.8)	0.828
NRS 2002 score	1.0 (0-1.00)	1 (0-1.75)	0.457
Barthel index	100 (95-100)	100 (95-100)	0.675

*Abbreviations*: *BMI* Body mass index, *NRS* Nutritional risk screening

**Table 2:** Prevalence of factors associated with the FRAX

Fracture-related factor	n (%)
Prior fragility fracture	25 (13.9)
Parental hip fracture	7 (3.9)
Smoking	28 (15.6)
Systemic glucocorticoid use	11 (6.1)
Rheumatoid arthritis	2 (1.1)
Other cases of secondary osteoporosis	16 (8.9)
Excess alcohol intake	20 (11.1)
Gender	
Female	115 (63.9)
Male	65 (36.1)

*Abbreviations*: *FRAX* Fracture Risk Assessment Tool

Using Image J software, we calculated the skeletal muscle and fat indices between the two groups and presented these indices in Table 3. SMI and PMI showed statistical differences between the two groups (84.91 versus 77.75 *p* = 0.024; 6.95 versus 5.90 *p* < 0.001). At the same time, VFI, SFI, and VSR were not statistically different between the two groups.

**Table 3:** Comparison of muscle and fat parameters between normal group and osteoporosis group

	Normal (112)	Osteoporosis(68)	*P* value
SMI (cm^2^/m^2^)	84.91 (75.78-95.36)	77.75 (67.97-90.65)	0.024
PMI (cm^2^/m^2^)	6.95 ± 1.69	5.90 ± 1.72	< 0.001
VFI (cm^2^/m^2^)	117.66 (90.92-134.60)	115.35 (94.50-129.01)	0.536
SFI (cm^2^/m^2^)	143.55 (127.43-168.05)	141.76 (128.57-168.84)	0.929
VSR	0.80 ± 0.21	0.78 ± 0.18	0.552

*Abbreviations*: *SMI* Skeletal muscle index, *PMI* Psoas muscle index, *VFI* Visceral fat index, *SFI* Subcutaneous fat index, *VSR* Visceral-to-subcutaneous ratio of fat area

### Correlation between BMD/FRAX score and indices (Table 4)

BMD of L1-4 was closely correlated with SMI, PMI, VFI and VSR (r = 0.199 *p* = 0.008, r = 0.422 *p* < 0.001, r = 0.253 *p* = 0.001, r = 0.310 *p* < 0.001). BMD of the femoral neck was only correlated with PMI and SFI (r = 0.268 *p* < 0.001, r = − 0.164 p-0.028). In addition, FRAX (major osteoporotic fracture) was only closely related to PMI (r = − 0.397 *p* < 0.001). FRAX (hip fracture) was closely related to SMI and PMI (r = − 0.183 *p* = 0.014, r = − 0.353 *p* < 0.001). Besides, FRAX (major osteoporotic fracture and hip fracture) did not correlate with VFI, SFI, and VSR.

**Table 4:** Correlation of BMD with SMI, PMI, SFI, VFI and VSR

	Spearman	*P* value
BMD (Lumbar spine1-4) (g/cm^2^)		
SMI (cm^2^/m^2^)	0.199	0.008
PMI (cm^2^/m^2^)	0.422	< 0.001
VFI (cm^2^/m^2^)	0.253	0.001
SFI (cm^2^/m^2^)	−0.094	0.211
VSR (cm^2^/m^2^)	0.310	< 0.001
BMD (Femoral neck) (g/cm^2^)		
SMI (cm^2^/m^2^)	0.075	0.321
PMI (cm^2^/m^2^)	0.268	< 0.001
VFI (cm^2^/m^2^)	0.002	0.976
SFI (cm^2^/m^2^)	−0.164	0.028
VSR (cm^2^/m^2^)	0.105	0.163
FRAX (major osteoporotic fracture), %		
SMI (cm^2^/m^2^)	−0.141	0.058
PMI (cm^2^/m^2^)	−0.397	< 0.001
VFI (cm^2^/m^2^)	−0.056	0.453
SFI (cm^2^/m^2^)	0.114	0.126
VSR (cm^2^/m^2^)	−0.139	0.062
FRAX (hip fracture), %		
SMI (cm^2^/m^2^)	−0.183	0.014
PMI (cm^2^/m^2^)	−0.353	< 0.001
VFI (cm^2^/m^2^)	−0.063	0.403
SFI (cm^2^/m^2^)	0.002	0.978
VSR (cm^2^/m^2^)	−0.067	0.369

*Abbreviations*: *BMD* Bone mineral density, *SMI* Skeletal muscle index, *PMI* Psoas muscle index, *VFI* Visceral fat index, *SFI* Subcutaneous fat index, *VSR* Visceral-to-subcutaneous ratio of fat area

### ROC analysis of indices in predicting osteoporosis

**Fig. 2:**
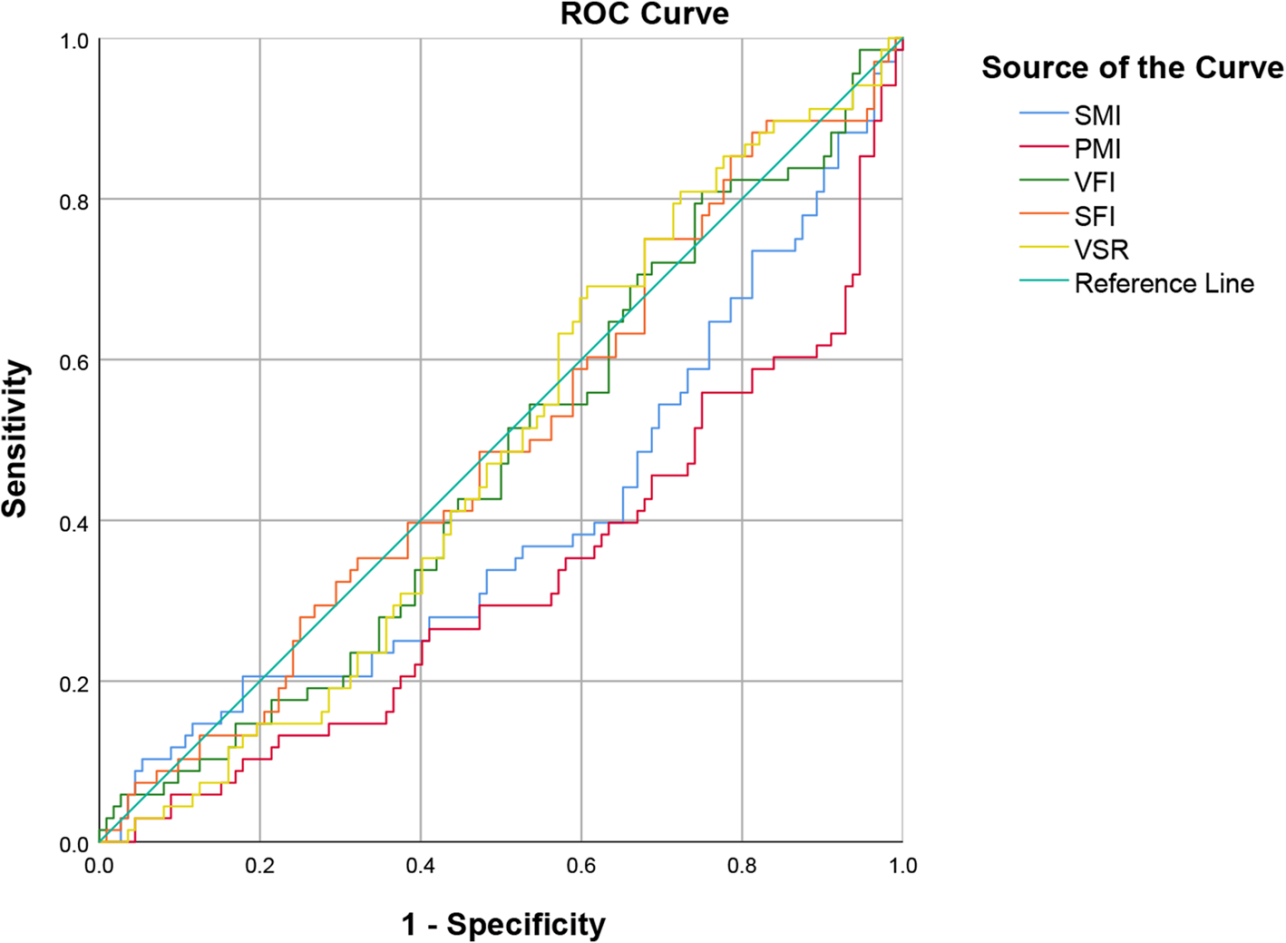
Receiver operating characteristic analysis of SMI, PMI, VFI, SFI and VSR ability to diagnose osteoporosis. Abbreviations: SMI, skeletal muscle index; PMI, psoas muscle index; VFI, visceral fat index; SFI, subcutaneous fat index; VSR, visceral-to-subcutaneous ratio of fat area

Table 5 shows the accuracy of SMI, PMI, VFI, SFI, and VSR in predicting osteoporosis. SMI and PMI were statistically significant, with the area under the curve (AUC) of 0.400 (95% confidence interval 0.312-0.488 *p* = 0.024) and 0.327 (95% confidence interval 0.244-0.410 *p* < 0.001), respectively. VFI, SFI, and VSR were not statistically significant in predicting osteoporosis. To better demonstrate the predictive efficacy of SMI, PMI, VFI, SFI, and VSR, the ROC graph is drawn in Fig. 2.

**Table 5:** Receiver operating characteristic curve analysis of muscle and fat parameters

	AUC	95% confidence interval	*P* value
SMI (cm^2^/m^2^)	0.400	0.312-0.488	0.024
PMI (cm^2^/m^2^)	0.327	0.244-0.410	< 0.001
VFI (cm^2^/m^2^)	0.472	0.386-559	0.559
SFI (cm^2^/m^2^)	0.496	0.409-0.583	0.929
VSR	0.482	0.397-0.567	0.693

*Abbreviations*: *AUC* Area under the curve, *SMI* Skeletal muscle index, *PMI* Psoas muscle index, *VFI* Visceral fat index, *SFI* Subcutaneous fat index, *VSR* Visceral-to-subcutaneous ratio of fat area

## Discussion

The results of this study are similar to those of previous studies [13, 19, 20], showing that skeletal muscle mass (SMI and PMI) can predict osteoporosis to some extent. However, contrary to previous studies [5, 14], VFI, SFI, and VSR measured on abdominal CT images at the L3 level were not effective predictors of osteoporosis in the present study. In addition, compared with SMI, PMI is closely correlated with BMD in the femoral neck and lumbar spine, and PMI has a more substantial predictive power. Similarly, PMI was more strongly associated with FRAX (major osteoporotic and hip fractures) than SMI, which partly means that PMI is a better predictor of osteoporosis and hip fractures over the next decade than SMI. Compared with skeletal muscle and adipose tissue at the same level, the L3 level of abdominal CT can better show the position and area of the psoas muscle [21, 22]. This indicates that PMI at the L3 level can better reflect the muscle mass of the whole body, which to some extent, explains the result of this study that PMI has better predictive ability than SMI, SFI, VFI, and VSR.

Many studies have explored the relationship between skeletal muscle and osteoporosis. The relationship between skeletal muscle and bone is not only mechanical. As endocrine organs, skeletal muscle and bone produce various cytokines, such as interleukin and irisin, which affect the growth and differentiation of osteogenic and osteoclast cells, thus affecting the function of bone and muscle [23]. Just as estrogen deficiency often causes osteoporosis, estrogen deficiency affects mitochondrial function in skeletal muscle cells, decreasing skeletal muscle mass and quantity [24]. As a classical signaling pathway, RANKL (Receptor activator of Nf-kb ligand) is closely related to the pathophysiological mechanism of osteoporosis. Bonnet, N. et al. [25] demonstrated that RANKL is closely associated with skeletal muscle function and that inhibition of RANKL activation can significantly improve muscle strength in patients with osteoporosis. These studies have revealed the relationship between skeletal muscle and osteoporosis at the cellular and molecular levels.

Similarly, several studies have demonstrated an association between skeletal muscle and osteoporosis at a clinical level. Recent studies have shown that skeletal muscle is closely related to osteoporosis. Low muscle mass is a risk factor for osteoporosis patients, and many experts encourage muscle strength training [26–28]. Several studies have shown that vitamin D and calcium are strongly associated with osteoporosis and muscle mass. As well as preventing osteoporosis, proper vitamin D and calcium supplementation can prevent muscle loss [29–31]. In elderly patients, low muscle mass often leads to unstable walking gait and osteoporotic fractures caused by falls [32, 33]. Several studies have demonstrated that low psoas mass is closely associated with low bone mass, osteoporosis, and osteoporotic fractures. Moreover, PMI can somewhat predict osteoporosis in patients with degenerative spinal diseases [20, 34]. Based on these studies, our team found that PMI had better predictive power for low bone mass, osteoporosis, and osteoporotic fractures than SMI, based on ROC and correlation analysis results.

Previous studies [14, 19] have shown a strong link between adipose tissue and osteoporosis. Therefore, VFI, SFI, and VSR indexes reflecting fat were also included in this study. However, to our surprise, VFI, SFI, and VSR were not effective predictors of osteoporosis. VFI, SFI, and VSR were also not strongly associated with FRAX (major osteoporotic and hip fractures). This may be because previous studies assessed osteoporosis by measuring the fat content of the whole body. However, this study only measured the fat area of the L3 level, which could not well measure the fat area of the liver and other essential organs. Therefore, the fat site at the L3 level alone is not an effective predictor of osteoporosis. Furthermore, a future study measuring the total abdominal fat area is needed further to explore the relationship between fat index and osteoporosis.

## Limitations

This study has the following advantages. First, the results of this study are consistent with those of similar previous studies, which significantly increases the reliability of the results of this study. Secondly, the variables of all participants in this study were complete, and participants with missing variables were excluded. Finally, most demographic and clinical baseline characteristics were not significantly different between normal and osteoporosis group participants. This makes the two groups of participants have certain comparability, reduces the bias, and provides support for the accuracy of the results of this study. However, this study has the following limitations. Firstly, although our team used a few methods to reduce the bias, this study is retrospective and prone to selection and recall bias. Secondly, this study only measured the area of skeletal muscle and adipose tissue at the L3 level. However, it did not measure the skeletal muscle and adipose tissue at the whole abdomen. This may cause the skeletal muscle and fat index at the L3 level not to reflect the entire body’s muscle mass and fat mass. Finally, relatively few participants in this study underwent DXA and abdominal CT within 3 months. Therefore, multi-center prospective studies with large sample sizes must be further studied.

## Conclusion

This study demonstrated that L3-based muscle index could assist clinicians in the diagnosis of osteoporosis to a certain extent, and PMI is superior to SMI in the diagnosis of osteoporosis. In addition, VFI, SFI, and VSR do not help clinicians to diagnose osteoporosis well.

## Data Availability

The datasets analyzed in the study are available from the corresponding author on reasonable request.

## Abbreviations

BMI: Body mass index
NRS: Nutritional risk screening
FRAX: Fracture Risk Assessment Tool
SMI: Skeletal muscle index
PMI: Psoas muscle index
VFI: Visceral fat index
SFI: Subcutaneous fat index
VSR: Visceral-to-subcutaneous ratio of fat area
BMD: Bone mineral density
AUC: Area under the curve
DXA: Dual-energy x-ray absorptiometry
CT: Computed tomography
L3: Lumbar spine 3
ROC: Receiver operating characteristic
